# Pulvinar quantitative susceptibility mapping predicts visual hallucinations post‐deep brain stimulation in Parkinson's disease

**DOI:** 10.1002/brb3.3263

**Published:** 2023-09-24

**Authors:** Keita Matsuura, Yuichiro Ii, Masayuki Maeda, Ken‐ichi Tabei, Masayuki Satoh, Maki Umino, Hiroyuki Kajikawa, Tommohiro Araki, Naoko Nakamura, Hirofumi Matsuyama, Akihiro Shindo, Hidekazu Tomimoto

**Affiliations:** ^1^ Department of Neurology, Graduate School of Medicine Mie University Mie Japan; ^2^ Department of Neuroimaging and Pathophysiology Mie University School of Medicine Mie Japan; ^3^ Department of Neuroradiology, Graduate School of Medicine Mie University Mie Japan; ^4^ School of Industrial Technology, Advanced Institute of Industrial Technology Tokyo Metropolitan Public University Corporation Tokyo Japan; ^5^ Department of Dementia and Neuropsychology, Advanced Institute of Industrial Technology Tokyo Metropolitan Public University Corporation Tokyo Japan; ^6^ Department of Radiology, Graduate School of Medicine Mie University Mie Japan; ^7^ Department of Neurology Suzuka Kaisei Hospital Mie Japan; ^8^ Department of Neurosurgery Suzuka Kaisei Hospital Mie Japan

**Keywords:** 3T magnetic resonance imaging, deep brain stimulation, Parkinson's disease, pulvinar nuclei, quantitative susceptibility mapping

## Abstract

**Purpose:**

We have reported the relationship between low pulvinar nuclei (PN) intensity in susceptibility‐weighted imaging and the appearance of visual hallucinations and cognitive function. The aim of the study was to examine the changes in the quantitative susceptibility mapping (QSM) in patients with Parkinson's disease (PD) who underwent deep brain stimulation (DBS) and verify whether the PN susceptibility value (SV) on QSM can predict visual hallucination and cognitive changes after DBS.

**Methods:**

This study examined 24 patients with PD who underwent DBS along with QSM imaging on magnetic resonance imaging (MRI). All MRIs were performed within 3 months before surgery. The PN SV was further assessed based on the QSM. Then, associations were examined among cognitive changes, hallucination, and PN SV. The cognitive function of the patient was compared immediately before surgery and at 1 year postoperatively.

**Results:**

Visual hallucinations were observed in seven patients during the follow‐up period. The PN SV was ≥0.045 ppm in nine patients with PD, and six of them had visual hallucinations, whereas only one of 15 patients with PD with SV of <0.045 ppm had visual hallucinations (Fisher's exact test, *p* = .0037).

**Conclusions:**

The SV of >0.045 ppm at the PN in QSM in patients with PD may provide useful information suggesting visual hallucination and cognitive deterioration after DBS treatment.

## Graphical abstract

In patients with Parkinson's disease 1 year after DBS, pulvinar nuclei in the QSM were associated with visual hallucination and cognitive worsening. We believe that this is useful information for the treatment and mechanisms of PD.

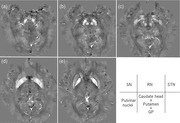

## 1 INTRODUCTION

Deep brain stimulation (DBS) is an established and beneficial therapy for motor fluctuation in patients with advanced Parkinson's disease (PD) (Krack et al., 2003). However, patients who undergo DBS may experience side effects, such as headache, seizures, difficulty in recalling, and postoperative deterioration of cognitive function (Heo et al., 2008). Brain edema associated with DBS in the perioperative period may contribute to these symptoms (Nishiguchi et al., 2022).

Patients with PD are presented not only with motor symptoms but also with various non‐motor symptoms. Occasionally, patients with PD may present with hallucinations during the course of the disease. Hallucinations are strongly associated with dementia (Marsili & Mahajan, 2022). PD has a background of α‐synuclein pathology, and PD and dementia with Lewy bodies (DLB) are diseases with a common pathological background (Jellinger, 2003). Some reports have indicated a relationship between pulvinar changes and DLB (Erskine et al., 2017; Watson et al., 2017; Yamamoto et al., 2006). Particularly, Erskine et al. (2017) reported that α‐synuclein was present throughout the pulvinar in DLB. Notably, α‐synuclein can bind Fe (II) and Fe (III) (Binolfi et al., 2006; Davies et al., 2011; Golts et al., 2002; Rao, 2007), and susceptibility‐weighted imaging (SWI) enables the evaluation of the tissues’ magnetic properties, such as blood or iron content (Sehgal et al., 2005). We have already reported the relationship between the low intensity of the pulvinar nucleus in SWI and the appearance of visual hallucinations after DBS surgery. After a 1‐year follow‐up of 21 subthalamic nucleus (STN)‐DBS cases, the group with hypointensity in the pulvinar nuclei in the prior SWI had a significantly higher frequency of visual hallucinations, including the transient ones. However, our previous study was based on the visual evaluation of hypointensity in pulvinar nuclei at SWI, and the evaluation may differ depending on imaging conditions (Matsuura et al., 2019). To avoid this ambiguity, we prefer a more quantitative evaluation of magnetic susceptibility, and we believe that quantitative susceptibility mapping (QSM) is an excellent method. QSM is a useful technique that quantitatively determines the bulk magnetic susceptibility distribution of tissue in vivo from gradient‐echo magnetic resonance phase images. Paramagnetic iron is commonly considered a predominant source of susceptibility variations in gray matter since many studies have reported a reasonable correlation between magnetic susceptibility and brain iron concentrations in vivo (Langkammer et al., 2012). The relationship between cognitive function and the pulvinar nuclei (PN), putamen (Put), and caudate nuclei has been reported in PD analysis using QSM (Langkammer et al., 2012; Shibata et al., 2022; Thomas et al., 2020).

This study aimed to evaluate the relationships between visual hallucination and the PN and the Put susceptibility value (SV) on QSM in patients with PD after DBS. Furthermore, we investigated the relationship between cognitive worsening and PN SV on QSM in these patients.

## 2 MATERIALS AND METHODS

### 2.1 Patients

This study was conducted retrospectively. Inclusion criteria were as follows: patients with PD who fulfilled the United Kingdom Brain Bank criteria (Hughes et al., 1992) and underwent DBS therapy at our institution from April 2017 to June 2019. A total of 24 patients (16 females and eight males; mean age: 62.7 years) matched these criteria. This study was approved by the institutional review boards of our institutions, and informed consent was provided by all patients before enrollment in the study.

### 2.2 MRI method

All patients underwent magnetic resonance imaging (MRI) at a 3T MRI unit (Ingenia; Philips Healthcare) with a 32‐channel head coil. All MRIs were performed within 3 months before surgery. The pulse sequence for QSM was a gradient‐echo SWI sequence using the following parameters: repetition time (TR), 25 ms; echo time (TE), 21 ms; field of view (FOV), 230 mm × 206 mm; matrix size, 320 × 250; slice thickness, 1.6 mm; and acquisition time, 4 min and 2 s. In this study, we used research software provided by FUJIFILM Healthcare Corporation. This software method consists of three steps: (I) iterative least square minimization, (II) adaptive edge‐preserving filtering to the susceptibility map in the minimization process, and (III) weighted addition of the susceptibility map in k‐space before and after filtering. This process was described in previous reports (Shirai et al., 2022, 2015). The pulse sequence for the T1‐weighted image (T1WI) was a three‐dimensional fast field‐echo technique using the following parameters: TR, 8.2 ms; TE, 4.6 ms; flip angles, 10; FOV, 260 mm; matrix size, 288 × 288; slice thickness, 0.9 mm; 200 slices; and acquisition time, 4 min and 42 s.

### 2.3 DBS method

All patients underwent bilateral electrode placement for STN DBS. Electrodes (Medtronic DBS lead models 3389; Medtronic and Vercise Cartesia DBS lead; Boston Scientific) were implanted under local anesthesia using a Leksell stereotactic frame (Elekta Instruments AB) and anatomical (MRI and computed tomography) and physiological targeting. Electrodes were considered correctly located in the target region based on microelectrode recordings. Impulse generators (Activa RC, Medtronic; Vercise Gevia) were implanted and connected during the second surgical procedure on the same day (Matsuura et al., 2019). The levodopa equivalent daily dose (LEDD) for each patient was calculated as follows: 100 mg L‐dopa/decarboxylase inhibitor = 1 mg pramipexole = 5 mg ropinirole = 3.3 mg/day rotigotine = 4 mg cabergoline = 70 mg L‐dopa/decarboxylase inhibitor with entacapone (Maschke et al., 2003; Parkin et al., 2002; Rabinak & Nirenberg, 2010).

### 2.4 QSM analysis

**FIGURE 1:**
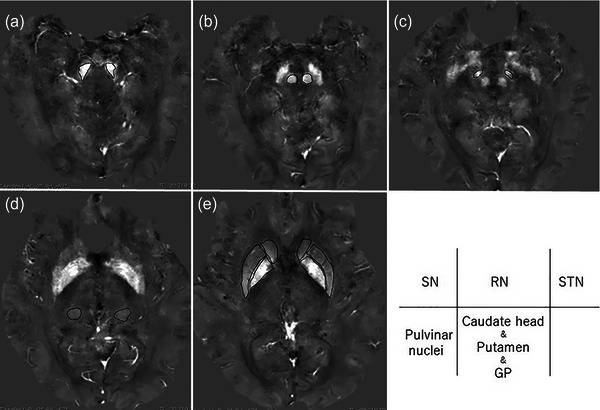
Quantitative susceptibility mapping (QSM) images; examples of the region of interest. Substantia nigra (a), red nucleus (b), subthalamic nucleus (c), pulvinar nucleus (d), caudate head, putamen, and globus pallidus (e). RN, red nucleus; SN, substantia nigra; STN, subthalamic nucleus; GP, globus pallidus.

The region of interest (ROI) of PN was set at the intercommissural (AC–PC) line slice. This ROI was determined in accordance with the Schaltenbrand and Wahren atlas (Schaltenbrand & Wahren, 1977) and our previous report (Matsuura et al., 2019). The ROI of STN was set at 6.4 mm below the AC–PC line in the QSM images. The ROI of substantia nigra (SN) was set at the slice of the red nucleus (RN) bottom. These ROIs were determined in accordance with the Schaltenbrand and Wahren atlas (Schaltenbrand & Wahren, 1977). The ROI of globus pallidus (GP), Put, and caudate head (Cau) were set at 3.2 mm above the AC–PC line. The ROI of RN was set to the slice with the largest longitudinal diameter. These ROIs were determined with reference to the Schaltenbrand and Wahren atlas (Schaltenbrand & Wahren, 1977) and a previous report (Barbosa et al., 2015) (Figure 1). Averaged SV in each ROI was measured. The process of these measurements was performed again by the same neurologist (Keita Matsuura) 1 year later and another neurologist (Naoko Nakamura) to evaluate intra‐ and inter‐rater reproducibility. The intraclass correlation coefficient (ICC) was calculated from these measurements. The SV of ROIs was taken from the first measurement of the neurologist (Keita Matsuura).

### 2.5 Neurological assessments

Assessments, including the Movement Disorder Society–Unified Parkinson's Disease Rating Scale (MDS‐UPDRS), Hoehn–Yahr stage, LEDD, Mini‐Mental State Examination (MMSE), the Japanese version of the Montreal Cognitive Assessment (MoCA‐J), the Trail Making Test (TMT), Raven's colored progressive matrices (RCPM), the Center for Epidemiologic Studies Depression Scale (CES‐D), and Pareidolia Test, were performed for all patients before DBS (i.e., baseline) and at 1 year postoperatively (Fujiwara et al., 2010; Goetz et al., 2007; Hoehn & Yahr, 1967; Lewinsohn et al., 1997; Folstein et al., 1975; Partington, 1949; Raven, 1941; Uchiyama et al., 2012). Additionally, the history of hallucinations was checked based on medical records. We asked patients and their caregivers whether they experienced hallucinations during hospitalization and outpatient visits. One patient was admitted to a different hospital for another disease after the first postoperative year, so it was not possible to evaluate the patient except for the presence of hallucinations. For this patient, the presence or absence of hallucinations at 1 year after surgery was confirmed by the patient's caregiver.

### 2.6 Statistical analyses

All statistical analyses except statistical power analysis were performed using the SPSS software (versions 26 and 29, IBM Corp.). The Wilcoxon signed‐rank tests were used to compare the Hoehn–Yahr stage, MDS‐UPDRS, MMSE, MoCA‐J, RCPM, Pareidolia test, and CES‐D between baseline and 1 year postoperatively. Additionally, paired *t*‐tests were used to compare the LEDD, L‐dopa dosage, and TMT‐A and TMT‐B between baseline and 1 year postoperatively. Moreover, the Mann–Whitney *U*‐test was used to compare MDS‐UPDRS, MMSE, MoCA‐J, RCPM, Pareidolia test, CES‐D, and Hoehn–Yahr stage between the groups with and without hallucination in the follow‐up period. An unpaired *t*‐test was performed to assess the SV between the groups with and without hallucination in the follow‐up period. Fisher's exact test was used to analyze hallucination and RCPM scores between SV values of ≥0.045 ppm and < 0.045 ppm of the PN in the QSM. Fisher's exact test was used to analyze hallucination and RCPM scores between SV of ≥0.085 ppm and < 0.085 ppm of the Put in the QSM. The Wilcoxon signed‐rank tests were used to compare the Hoehn–Yahr stage, MDS‐UPDRS, MMSE, MoCA‐J, RCPM, Pareidolia test, and CES‐D between the PN SV of ≥0.045 ppm and < 0.045 ppm in the QSM. Paired *t*‐tests were used to compare the LEDD, L‐dopa dosage, and TMT‐A and TMT‐B between the PN SV of ≥0.045 ppm and <0.045 ppm in the QSM. Inter‐ and intra‐rater reliabilities of the SV of ROI on QSM were measured using two‐way random average measure and one‐way random single measure ICC and associated 95% confidence intervals (CIs). We performed multivariable analysis using a generalized linear model. As a predictor variable, we used the history of hallucination, the Put SV, and the PN SV. Receiver operating characteristic (ROC) curves were constructed to assess the sensitivity and specificity of the pulvinar or putamen SV in predicting postoperative hallucination. Cohen's *d* was calculated by G*Power (Faul et al., 2009, 2007).

## 3 RESULTS

During the DBS operation, the patients’ average age (mean ± standard deviation) was 62.7 ± 8.7 years. Additionally, the average motor performance of off state (Hoehn–Yahr stage and MDS‐UPDRS part III) significantly improved and LEDD and L‐dopa dosage significantly decreased 1 year postoperatively (Table 1). The MMSE and CES‐D scores at 1 year were significantly better than preoperatively (*p* = .048, .002, respectively). Other cognitive performance tests remained unchanged (Table 1).

**TABLE 1:** Demographic data and overall result.

	Pre‐operation	After 1 year^a^
Age	62.7 ± 8.7	
Sex (M:F)	8:16	
Disease duration (years)	13.4 ± 6.2	
DBS target	All STN	
Hoehn–Yahr stage (on state)	2.6 ± 0.9	2.4 ± 0.7
Hoehn–Yahr stage (off state)	4.4 ± 0.6	2.8 ± 0.5**
MDS‐UPDRS Part I	9.5 ± 4.4	7.7 ± 4.6*
MDS‐UPDRS Part II	10.4 ± 6.3	7.9 ± 4.0
MDS‐UPDRS Part III on/off	17.3 ± 11.7/42.8 ± 18.5	12.7 ± 8.8/16.4 ± 13.0**
MDS‐UPDRS Part IV	9.7 ± 5.0	4.1 ± 3.5**
LEDD (mg)	897 ± 287	558 ± 238**
l‐Dopa (mg)	470 ± 145	312.5 ± 112**
DA (use rate; %)	91.7	62.5
Entacapone (use rate, %)	75	62.5
Selegiline (use rate, %)	33.3	20.8
Rasagiline (use rate, %)	0	16.7
Zonisamide (use rate, %)	29.2	45.8
Istradefylline (use rate, %)	41.7	54.2
MMSE	28.3 ± 1.6	29.1 ± 1.5*
MoCA‐J	26.2 ± 2.6	26.9 ± 1.8
RCPM	30.3 ± 4.1	29.0 ± 4.4
TMT‐A (s)	130.8 ± 55.7	115.2 ± 59.9
TMT‐B (s)	208.5 ± 132.1	165.2 ± 98.6
Pareidolia test (%)	4.3 ± 11.4	0.22 ± 0.72
CES‐D	16.6 ± 7.6	10.2 ± 6.5**

Abbreviations: CES‐D, Center for Epidemiologic Studies Depression Scale; DA, dopamine agonist; DBS, deep brain stimulation; F, female; LEDD, levodopa equivalent daily dose; M, male; MDS, Movement Disorder Society; MMSE, Mini‐Mental State Examination; MoCA‐J, Japanese version of Montreal Cognitive Assessment; RCPM, Raven's Colored Progressive Matrices; STN, subthalamic nucleus; TMT, Trail Making Test; UPDRS, Unified Parkinson's Disease Rating Scale.

^a^Evaluation of 23 cases.

*p < .05; **p < .01.

Postoperative visual hallucinations (H+) were determined in seven cases but not 17 cases (H−). The type of hallucination was both visual and auditory in only one case and only visual in six cases. In only one case, hallucinations persisted 6 months after DBS. In the other six patients, hallucinations were transient, occurring three times in one case and only once in all other cases. At the time of preoperative evaluation, no patient experienced hallucinations. Among seven cases with postoperative hallucinations, five had a history of hallucination (including drug‐derived hallucinations); among the other 17 cases, only two had a history of hallucination. Cohen's *d* for SV among groups with and without hallucinations was 1.695.

**FIGURE 2:**
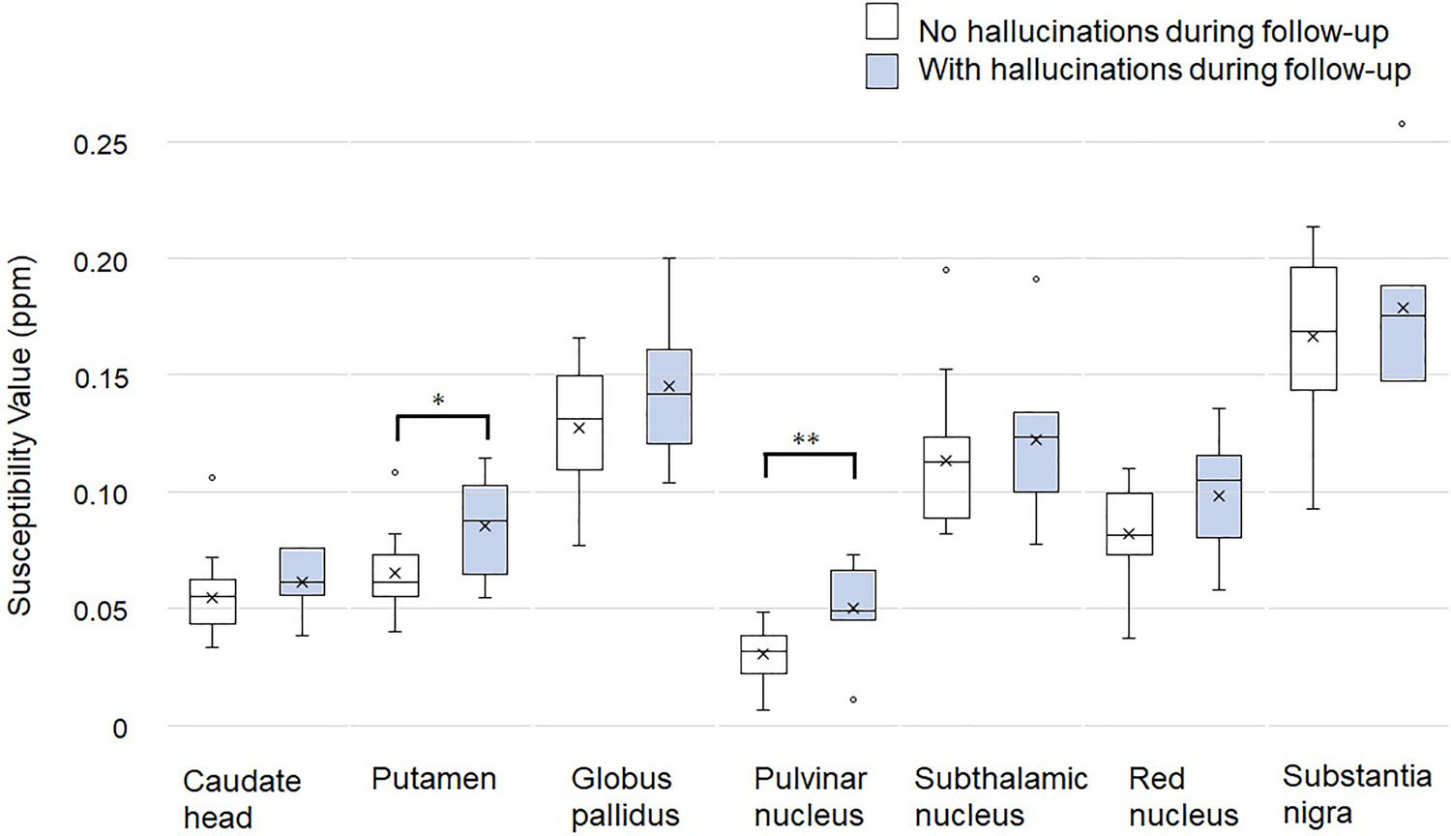
All results are expressed as box‐and‐whisker plots. The plots represent the comparison of the susceptibility value of each target between groups with and without hallucinations during follow‐up. **p < .01; *p < .05.

The PN and Put SV was 0.0502 ± 0.0076 and 0.0854 ± 0.0078 ppm in the H+ group and 0.0305 ± 0.0030 and 0.0650 ± 0.0038 ppm in the H− group, respectively (Student's *t*‐test, *t* (22) = −2.960, *p* = .007; *t* (22) = −2.640, 0.015, respectively). No significant difference was found in the QSM image SV in other nuclei between the H+ and H− groups (Table 2, Figure 2).

**TABLE 2:** Differences in the presence or absence of hallucinations during the follow‐up period.

	Pre‐operation		After 1 year^a^		Amount of change over 1 year^a^	
Hallucination during the follow‐up period	Occurred	Nothing	Occurred	Nothing	Occurred	Nothing
Age	62.6+9.2	63.0+7.8	–	–	–	–
Sex (M:F)	1:6	7:10	–	–	–	–
Disease duration (years)	13.7+7.3	12.8+2.7	–	–	–	–
History of hallucinations	5	2	–	–	–	–
The SV on QSM image						
Caudate head (ppm)	0.0636 ± 0.0055	0.0548 ± 0.0049	–	–	–	–
Putamen (ppm)	0.0854 ± 0.0078	0.0650 ± 0.0038 *	–	–	–	–
Globus pallidus (ppm)	0.1455 ± 0.0115	0.1272 ± 0.0063	–	–	–	–
Pulvinar nucleus (ppm)	0.0502 ± 0.0076	0.0305 ± 0.0030 **	–	–	–	–
Subthalamic nucleus (ppm)	0.1225 ± 0.0136	0.1135 ± 0.0071	–	–	–	–
Red nucleus (ppm)	0.0982 ± 0.0098	0.0822 ± 0.0047	–	–	–	–
Substantia nigra (ppm)	0.1785 ± 0.0145	0.1665 ± 0.0086	–	–	–	–
Hoehn–Yahr stage (on)	2.9 ± 0.9	2.5 ± 0.9	2.9 ± 0.7	2.2 ± 0.6*	0.0 ± 0.6	−0.3 ± 0.6
Hoehn–Yahr stage (off)	4.9 ± 0.4	4.2 ± 0.6*	3.1 ± 0.4	2.7 ± 0.5*	−1.6 ± 0.5	−1.7 ± 0.5
MDS‐UPDRS Part I	9.0 ± 2.8	9.8 ± 5.0	7.9 ± 4.5	7.6 ± 4.9	−1.1 ± 3.7	−2.1 ± 4.0
MDS‐UPDRS Part II	11.3 ± 5.5	9.6 ± 6.5	8.7 ± 5.3	7.5 ± 3.4	−2.6 ± 6.1	−1.8 ± 7.4
MDS‐UPDRS Part III on	17.6 ± 12.1	17.1 ± 12.0	16.1 ± 11.4	11.2 ± 7.3	−1.4 ± 6.1	−6.3 ± 14.1
MDS‐UPDRS Part III off	46.0 ± 19.7	41.5 ± 18.4	20.1 ± 20.3	14.6 ± 8.1	−25.9 ± 21.6	−27.1 ± 21.1
MDS‐UPDRS Part IV	9.9 ± 3.7	9.6 ± 5.6	4.1 ± 3.4	4.1 ± 3.6	−5.7 ± 5.2	−5.4 ± 6.0
LEDD (mg)	869 ± 115	909 ± 336	496 ± 226	584 ± 245	−373 ± 199	−325 ± 264
l‐Dopa (mg)	450 ± 58	477 ± 174	271 ± 104	329 ± 114	−179 ± 111	−149 ± 149
MMSE	28.4 ± 28.2	28.2 ± 1.6	28.7 ± 1.5	29.3 ± 1.6	0.3 ± 1.0	1.0 ± 2.3
MoCA‐J	25.6 ± 26.4	26.4 ± 2.5	25.7 ± 2.1	27.4 ± 1.5	0.1 ± 2.6	1.2 ± 2.7
RCPM	29.1 ± 6.3	30.7 ± 2.9	27.1 ± 5.2	29.9 ± 3.9	−2.0 ± 3.7	−0.8 ± 3.0
TMT‐A (s)	161.4 ± 65.7	118.1 ± 47.6	143.7 ± 82.2	102.7 ± 44.8	−17.7 ± 71.9	−15.1 ± 44.1
TMT‐B (s)	236.3 ± 124.1	197.0 ± 137.3	219.0 ± 153.4	141.6 ± 53.8	−17.3 ± 130.2	−57.4 ± 109.2
Pareidolia test (%)	5.0 ± 12.2	4.0 ± 11.4	0	0.3 ± 0.9	−5.0 ± 12.2	−3.9 ± 11.8
Apathy scale	9.6 ± 6.9	12.5 ± 7.2	9.9 ± 2.4	13.9 ± 6.9	0.3 ± 6.6	1.8 ± 6.5
CES‐D	17.7 ± 8.2	16.1 ± 7.6	9.9 ± 9.2	10.4 ± 5.3	−7.9 ± 6.3	−4.9 ± 7.5

Abbreviations: CES‐D, Center for Epidemiologic Studies Depression Scale; DA, dopamine agonist; DBS, deep brain stimulation; F, female; LEDD, levodopa equivalent daily dose; M, male; MDS, Movement Disorder Society; MMSE, Mini‐Mental State Examination; MoCA‐J, Japanese version of Montreal Cognitive Assessment; QSM, quantitative susceptibility mapping; RCPM, Raven's Colored Progressive Matrices; STN, subthalamic nucleus; SV, susceptibility value; TMT, Trail Making Test; UPDRS, Unified Parkinson's Disease Rating Scale.

^a^ Evaluation of 23 cases.

*p < .05; **p < .01.

The PN SV was ≥0.045 ppm in nine patients with PD, six of them had visual hallucinations, whereas only one of 15 patients with PD with PN SV of <0.045 ppm had visual hallucinations (Fisher's exact test, *p* = .0037) (Table 3).

**TABLE 3:** Relationship between cognitive function and pulvinar or putamen susceptibility value (SV) in quantitative susceptibility mapping (QSM) image.

	Hallucination occurred during the follow‐up period	
SV in QSM image	Nothing	Occurred
Pulvinar nucleus		
≥0.045 ppm	3	6
<0.045 ppm	14	1
		*p* = .0037
Putamen		
≥0.085 ppm	1	4
<0.085 ppm	16	3
		*p* = .0014

The Put SV was ≥0.085 ppm in five patients with PD, and four of them had visual hallucinations. Out of 19 patients with PD with SV of <0.085 ppm, three had visual hallucinations (Fisher's exact test, *p* = .014) (Table 3).

We performed a multivariable analysis of three factors for the appearance of postoperative hallucinations: history of hallucinations, SV of PN, and SV of Put. The standardized partial regression coefficient for the history of hallucinations, SV of Put, and SV of PN was 0.547 (95% CI: 0.432–0.693; *p* < .001), 1.006 (95% CI: 1.001–1.012; *p* = .031), and 1.009 (95% CI: 1.003–1.016; *p* = .006), respectively (Table 4 ).

**TABLE 4:** Factors on post‐deep brain stimulation (DBS) hallucination occurrence were evaluated with multivariable analysis using a generalized linear model.

	SPRC	95% CI	*p*
History of hallucinations	0.547	0.432–0.693	<.001
The SV of putamen on QSM	1.006	1.001–1.012	.031
The SV of pulvinar nucleus on QSM	1.009	1.003–1.016	.006

Abbreviations: CI, confidence interval; QSM, quantitative susceptibility mapping; SPRC, standardized partial regression coefficient; SV, susceptibility value.

**FIGURE 3:**
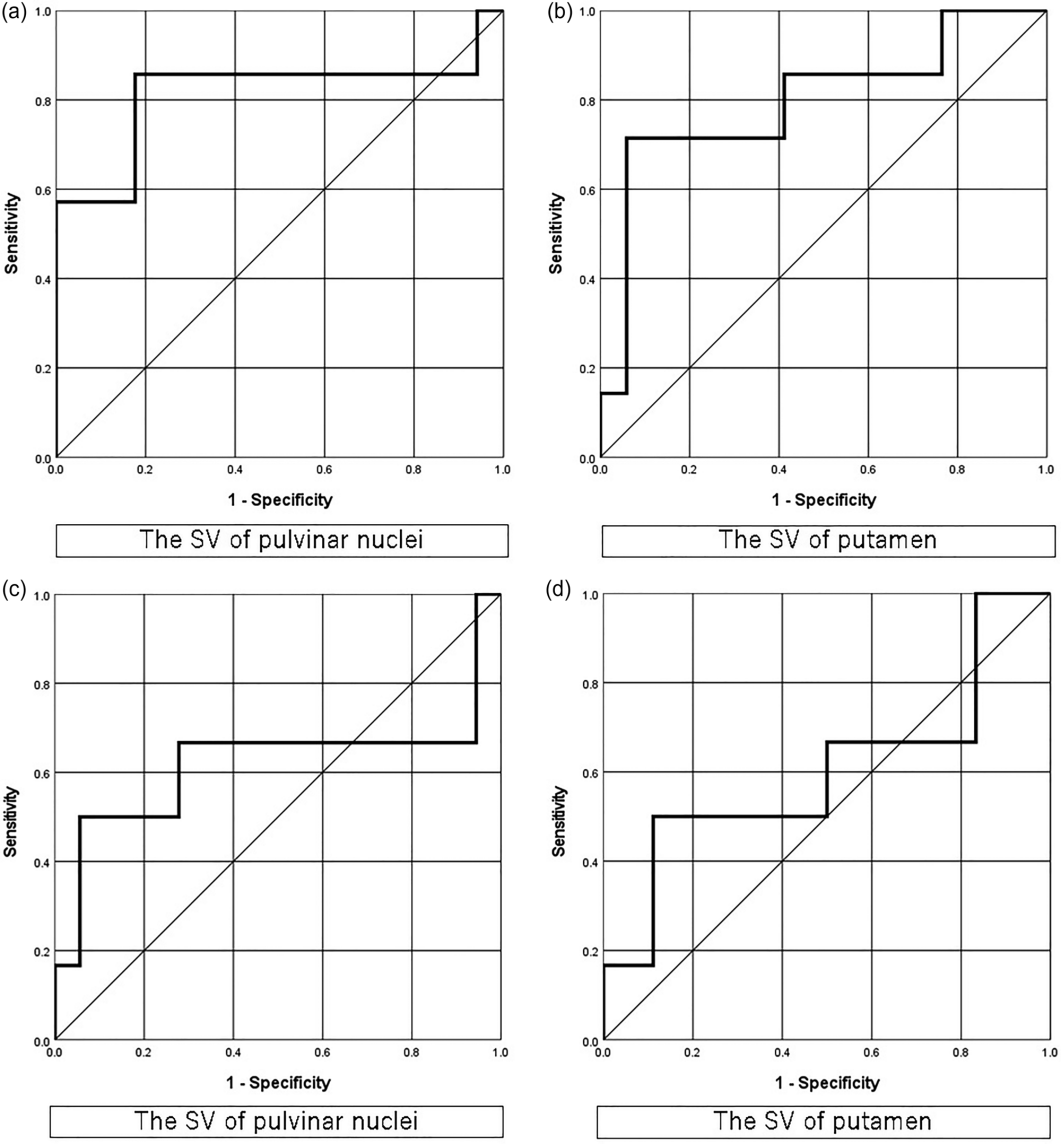
Receiver operating characteristic curves were constructed to predict post (a and b) and preoperative hallucination (c and d). The area under the curve was 0.82 (95% confidence interval [CI]: 0.57−1.00) at the pulvinar nuclei susceptibility value (SV) and 0.80 (95% CI: 0.58−1.00) at the putamen SV.

Using ROC curves to predict postoperative hallucination, the area under the curve (AUC) was 0.82 (95% CI: 0.57−1.00, *p* = .012) at the PN SV and 0.80 (95% CI: 0.58−1.00, *p* = .024) at the Put SV. Using ROC curves to predict preoperative hallucination, the AUC was 0.60 (95% CI: 0.30−0.90, *p* = .39) at the PN SV and 0.62 (95% CI: 0.28−0.96, *p* = .46) at the Put SV (Figure 3).

Of seven patients with visual hallucination, six had >0.045 ppm of the PN SV, four had >0.085 ppm of the Put SV, three patients met both PN and Put, three patients met only PN, one patient met only Put, and no patient met none. The PN SV in QSM images was compared between the groups of ≥0.045 ppm and <0.045 ppm, and a significant difference was found in age between the groups of ≥0.045 ppm and <0.045 ppm, 57.6 ± 2.7 years and 65.8 ± 7.7 years, respectively (*p* = .020). A significant difference was also found in RCPM worsening over 1 year at 2.9 ± 2.5 in the SV of >0.045 ppm and 0.0 ± 3.1 in <0.045 ppm (*p* = .025). No other significant differences were observed in the assessment of MDS‐UPDRS, Hoehn–Yahr stage, and cognition (Table 5).

**TABLE 5:** Comparison between two groups with the susceptibility value (SV) of pulvinar nucleus in quantitative susceptibility mapping (QSM) above and below 45 ppm.

	Pre‐operation		After 1 year^a^		Amount of change over one year^a^	
The SV of pulvinar nucleus on QSM	≥45 ppm	<45 ppm	≥45 ppm	<45 ppm	≥45 ppm	<45 ppm
Age	57.6 ± 2.7	65.8 ± 7.7*	–	–	–	–
Sex (M:F)	4:5	4:11	–	–	–	–
Disease duration (years)	14.7 ± 9.1	12.7 ± 3.9	–	–	–	–
Hoehn–Yahr stage (on state)	2.6 ± 0.9	2.5 ± 0.9	2.4 ± 0.5	2.3 ± 0.8	−0.2 ± 0.8	−0.2 ± 0.4
Hoehn–Yahr stage (off state)	4.7 ± 0.5	4.3 ± 0.6	2.9 ± 0.3	2.8 ± 0.6	−1.8 ± 0.4	−1.5 ± 0.5
MDS‐UPDRS Part I	11.3 ± 4.8	8.5 ± 4.0	8.8 ± 4.9	6.9 ± 4.5	−2.6 ± 4.4	−1.4 ± 3.5
MDS‐UPDRS Part II	10.0 ± 5.9	10.1 ± 6.5	8.0 ± 4.5	7.8 ± 3.8	−2.0 ± 6.1	−2.1 ± 7.6
MDS‐UPDRS Part III on	19.4 ± 13.3	15.9 ± 10.9	12.4 ± 9.5	12.9 ± 8.7	−7.0 ± 12.7	−3.4 ± 12.3
MDS‐UPDRS Part III off	48.7 ± 20.7	39.3 ± 16.8	15.9 ± 18.2	16.7 ± 8.6	−32.8 ± 21.9	−22.5 ± 19.7
MDS‐UPDRS Part IV	10.1 ± 5.4	9.5 ± 4.9	3.0 ± 3.0	4.9 ± 3.7	−7.1 ± 6.1	−4.4 ± 5.3
LEDD (mg)	917 ± 174	884 ± 342	521 ± 201	580 ± 262	−396 ± 281	−305 ± 222
l‐Dopa (mg)	467 ± 83	471 ± 179	291 ± 95	325 ± 122	−175 ± 113	−147 ± 153
MMSE	28.0 ± 1.6	28.5 ± 1.6	29.0 ± 1.4	29.1 ± 1.7	1.0 ± 1.9	0.6 ± 2.1
MoCA‐J	25.4 ± 2.2	26.6 ± 2.9	26.7 ± 1.9	27.0 ± 1.8	1.2 ± 3.2	0.6 ± 2.4
RCPM	30.8 ± 3.6	29.9 ± 4.4	27.9 ± 4.2	29.8 ± 4.5	−2.9 ± 2.5	0.0 ± 3.1*
TMT‐A (s)	159.4 ± 69.7	113.6 ± 38.4	130.6 ± 72.2	105.3 ± 50.9	−28.9 ± 75.1	−7.5 ± 31.1
TMT‐B (s)	200.9 ± 89.1	213.0 ± 155.2	192.2 ± 138.8	147.8 ± 61.3	−8.7 ± 103.5	−68.7 ± 118.7
Pareidolia test (%)	0.3 ± 0.8	6.7 ± 14.0	0 ± 0	0.4 ± 0.9	−0.3 ± 0.8	−6.8 ± 14.6
Apathy scale	9.9 ± 6.4	12.7 ± 7.5	12.3 ± 8.1	12.9 ± 4.8	2.4 ± 8.2	0.6 ± 5.2
CES‐D	17.7 ± 6.9	15.9 ± 8.2	9.9 ± 9.9	10.4 ± 3.4	−7.8 ± 6.9	−4.6 ± 7.2

Abbreviations: CES‐D, Center for Epidemiologic Studies Depression Scale; DA, dopamine agonist; DBS, deep brain stimulation; F, female; LEDD, levodopa equivalent daily dose; M, male; MDS, Movement Disorder Society; MMSE, Mini‐Mental State Examination; MoCA‐J, Japanese version of Montreal Cognitive Assessment; QSM, quantitative susceptibility mapping; RCPM, Raven's Colored Progressive Matrices; STN, subthalamic nucleus; SV, susceptibility value; TMT, Trail Making Test; UPDRS, Unified Parkinson's Disease Rating Scale.

^a^ Evaluation of 23 cases.

*p < .05; **p < .01.

The SV analyses revealed the intra‐rater ICC for the SN, RN, STN, Put, GP, PN, and Cau as 0.78, 0.89, 0.80, 0.93, 0.93, 0.88, and 0.93, respectively (95% CI: 0.56−0.90, 0.76−0.95, 0.59−0.91, 0.86−0.97, 0.84−0.97, 0.74−0.94, and 0.84−0.97, respectively, all *p* < .001), whereas the inter‐rater ICC was 0.68, 0.89, 0.86, 0.91, 0.90, 0.73, and 0.73, respectively (95% CI: 0.38−0.85, 0.77−0.95, 0.71−0.94, 0.70−0.97, 0.76−0.96, 0.45−0.88, and 0.47−0.87, respectively, all *P* < 0.001).

## 4 DISCUSSION

This study revealed that the SV of PN and Put in QSM was associated with experiencing visual hallucinations during the first year after DBS surgery. Additionally, a relationship was found between PN SV and RCPM worsening. These results are similar to our previous study (Matsuura et al., 2019). Our previous study used SWI to distinguish whether the pulvinar could be seen as low signal or not, whereas the present study used the PN and Put SV in QSM as the index of visual hallucination. The QSM was quantifiable, with a threshold of 0.045 ppm for PN SV and 0.082 ppm for Put (Langkammer et al., 2012; Shibata et al., 2022). A higher rate of visual hallucination occurrence is found in cases that are more elevated than those values. PN is strongly associated with the occipital lobe (Benarroch, 2015). In DLB, α‐synuclein deposition is also observed in the PN (Erskine et al., 2017). α‐Synuclein can bind Fe (II) and Fe (III) (Binolfi et al., 2006; Davies et al., 2011; Golts et al., 2002; Rao, 2007). Analysis of the ROC curve predicting postoperative hallucinations showed that the AUC of PN was as high as 0.82. Therefore, the association between the high PN SV and the appearance of visual hallucination is reasonable. However, the confidence interval is large (0.57–1.00), so further accumulation of cases is needed. On the other hand, AUC values were lower in the ROC curves between the history of visual hallucinations and SV of PN and Put in QSM. This result may be due to the insufficient number of cases.

Recently, the relationship between brain iron deposition assessed by QSM and cognitive severity in PD was reported. The lower MoCA score revealed increased brain tissue iron in the hippocampus, thalamus, Put, and caudate nucleus (Thomas et al., 2020). Meanwhile, Put has been associated with auditory hallucinations in fMRI studies (Cui et al., 2016; Salisbury et al., 2021). However, no reports have demonstrated a direct association between Put and visual hallucinations in patients with PD.

However, the appearance of postoperative hallucinations was related to the SV of PN and Put and the history of preoperative hallucinations in the multivariable analysis. More factors need to be examined to predict the occurrence of hallucinations more accurately, and further studies with larger numbers of cases are needed.

Seed‐based analyses in patients with DLB with visual hallucination reported decreased connectivity of the lateral geniculate nucleus, the superior parietal lobule, and the Put with the medial frontal gyrus (Pezzoli et al., 2021), and whole‐brain voxel‐based morphometry analysis revealed decreased gray matter volume in DLB with visual hallucination in the right superior and medial frontal gyri, Put, caudate nucleus, and insula (Pezzoli et al., 2019), which suggested that Put may have an important role in visual function. Alternatively, it may simply reflect the spread of the pathology. One year later, RCPM was significantly worse in the group with higher PN SV; the results may be related to the strong association between the PN and the occipital lobe since RCPM is a mandatory visual processing test (Abe et al., 2003; Benarroch, 2015).

As mentioned above, caudate nuclei have also been reported to be associated with cognitive function. In the present study, however, no significant changes were found in the occurrence of visual hallucinations. However, there was a slightly higher tendency in the hallucination occurrence group, so the results may not have been clear due to the insufficient number of cases.

The results of this study indicate a significant improvement in MMSE scores obtained after 1 year of receiving DBS treatment compared with those obtained preoperatively. As PD is accompanied by fluctuating symptoms, preoperative evaluations are often performed when patients are in their ON state. However, due to the unpredictable nature of these symptoms, it is not always possible to evaluate optimal conditions. Notably, research has shown that patients experience slowness of thinking in their OFF state, suggesting that these effects may still influence the evaluation outcomes (Witjas et al., 2002).

The present study had several limitations. First, the ROIs of PN and other targets were assessed freehand. Future studies should confirm and extend our findings using the auto‐segmented method. Second, this study has a small sample size, and the follow‐up period is not long enough. Therefore, further study is needed. Third, hallucinations were evaluated only by conducting inpatient and outpatient interviews. In the future, it will be necessary to use well‐validated evaluation criteria such as Neuropsychiatric Inventory (Cummings et al., 1994). Fourth, many factors are involved in visual hallucinations and cognitive function; some items are interrelated. Therefore, further case studies are needed to interpret the exact meaning of each factor, which is a task for the future. Fifth, to increase the reliability of this study, it would be desirable to add a third‐party analysis of images and statistics, and we believe this is an issue for future research.

In conclusion, the SV of ≥0.045 ppm at the PN in patients with PD may provide information that is useful for suggesting visual hallucination and cognitive deterioration after DBS treatment.

## AUTHOR CONTRIBUTIONS

Keita Matsuura, Yuichiro Ii, Masayuki Maeda, Akihiro Shindo, and Hidekazu Tomimoto helped with the conception of the study. Keita Matsuura, Hiroyuki Kajikawa, and Naoko Nakamura helped with the organization of the study. Keita Matsuura, Tommohiro Araki, and Hiroyuki Kajikawa executed the study. In terms of statistical analyses, Ken‐ichi Tabei and Keita Matsuura designed, Ken‐ichi Tabei executed, and Hiroyuki Kajikawa and Masayuki Satoh helped with the review and critique. In terms of QSM analyses, Keita Matsuura and Masayuki Maeda designed; Maki Umino, Keita Matsuura, and Ken‐ichi Tabei executed; and Masayuki Maeda helped with the review and critique. Keita Matsuura drafted the first manuscript, and Yuichiro Ii, Masayuki Maeda, and Hidekazu Tomimoto helped with the review and critique.

## CONFLICT OF INTEREST STATEMENT

Ken‐ichi Tabei and Masayuki Satoh are employed by Tokyo Metropolitan Public University Corporation. The remaining authors declare no conflicts of interest.

### PEER REVIEW

The peer review history for this article is available at https://publons.com/publon/10.1002/brb3.3263.

## Data Availability

The data generated and analyzed in this study are available from the corresponding author upon reasonable request.
